# PReS-FINAL-2074: The role of the probiotic VSL-3 as adjuvant therapy in patients with undifferentiated spondyloarthritis (ERA)

**DOI:** 10.1186/1546-0096-11-S2-P86

**Published:** 2013-12-05

**Authors:** M Vidovic, M Perica, L Lamot, M Zaninovic, L Tambic Bukovac, M Harjacek

**Affiliations:** 1 Childrens Hospital Srebrnjak, Zagreb, Croatia

## Introduction

Calprotectin is a neutrophil derived protein that binds calcium and belongs to the S100 family. It can be quantified in feces and has become established as a marker of gut inflammation, where increased levels are a direct result of neutrophil migration into the gut lumen across the inflamed mucosa. Since subclinical gut inflammation is present in the majority of adult and pediatric spondyloarthritis patients, fecal calprotectin (fcal) is emerging as possible noninvasive biomarker. There is strong evidence supporting the role of the VSL-3 probiotic in decreasing fcal, thus promoting and maintaining remission in patients with inflammatory bowel disease (IBD), but very little is known of its potential effects on disease activity in children with undifferentiated spondyloarthritis (ERA).

## Objectives

To assess the effect of the VSL-3 probiotic on fcal levels, clinical symptoms and disease activity of children with undifferentiated spondyloarthritis (ERA).

## Methods

Sixteen patients diagnosed with ERA, according to the ILAR criteria, were treated with VSL-3, in addition to standard therapy (NSAID's and DMARD's). All patients were negative for gastrointestinal (GI) symptoms, and/or poor growth. All four ankylosing spondylitis patients received biologics; two were treated with adalimumab and further two with infliximab. In addition to general clinical data, patients completed the BASFI and BASDAI questioners, ESR and CRP were obtained, and fcal was measured by the Calprest ELISA method (Eurospital Spa, Italy). After VSL-3 was introduced, two follow up visits were scheduled; the first after one month and the second after three months. At the three month visit a second fcal value was obtained, and BASFI and BASDAI were reevaluated.

## Results

The baseline mean fcal level was 52,3 mg/kg (normal < 50 mg/kg). Nine patients have completed all scheduled visits to date, and eight out of those nine had markedly decreased fcal levels (mean value 15,6 mg/kg). The BASFI index was decreased in seven patients and the BASDAI index in eight patients. At the follow-up visit, none of the patients were found to have developed GI symptoms or other signs and symptoms suggestive of IBD.

## Conclusion

VSL-3 is emerging as a possible potent adjuvant therapy in children and adults with IBD thus encouraging its use in patients with ERA. To our knowledge our study is the first in evaluating the effects of VSL-3, as adjuvant therapy, on disease activity in ERA patients. Preliminary data suggest that the use of VSL-3 can decrease fcal levels, and together with standard therapy can improve clinical symptoms and decrease disease activity in patients with ERA. A larger patient cohort is needed to confirm VSL-3 efficacy in ERA patients.

## Disclosure of interest

None declared.

